# Reverse Cervical Lordosis Caused by Giant Vertebral Artery Aneurysm in von Recklinghausen Disease

**DOI:** 10.7759/cureus.29795

**Published:** 2022-09-30

**Authors:** Umaira Saleh, Muhammad Ihfaz Ismail, Nur Asma Sapiai, Kok Beng Loh, Nasser Abd Wahab, Jafri M Abdullah

**Affiliations:** 1 Department of Neuroscience, School of Medical Sciences, Universiti Sains Malaysia, Kubang Kerian, MYS; 2 Department of Neuroscience, Universiti Sains Malaysia (USM) Health Campus, Hospital Universiti Sains Malaysia, Kubang Kerian, MYS; 3 Department of Neurosurgery, Hospital Pulau Pinang, Georgetown, MYS; 4 Department of Radiology, School of Medical Sciences, Universiti Sains Malaysia, Kubang Kerian, MYS; 5 Department of Neuroscience, Brain and Behaviour Cluster, Universiti Sains Malaysia, Kubang Kerian, MYS; 6 Department of Radiology, Hospital Pulau Pinang, Georgetown, MYS

**Keywords:** pseudoaneurysm, reverse cervical lordosis, endovascular embolization, vertebral artery aneurysm, neurofibromatosis

## Abstract

Neurofibromatosis type 1 (NF1) is a variable penetrance autosomal dominant condition predominantly involving the peripheral nervous system. NF1 exhibits a wide spectrum of clinical patterns involving the skin, eye, brain, spinal cord, and, to a lesser extent, long bones and arteries. Arterial stenosis or aneurysms have been variously studied, but the association with NF1 has not been firmly established. A 31-year-old gentleman with NF1 experienced progressive neck pain over a five-month period, associated with limited range of motion and dysphagia. Magnetic resonance imaging (MRI) of the cervical spine suggests paraspinal plexiform neurofibromas with excessive reverse cervical lordosis. Further workups revealed a large left vertebral artery fusiform aneurysm and a pseudoaneurysm. The patient made a full recovery following endovascular embolization. It is crucial to maintain a high index of suspicion for vascular malformations in patients with NF1. The pathogenesis of vascular manifestations in NF1 and options for therapeutic management were discussed.

## Introduction

Neurofibromatosis type 1 (NF1) is an autosomal dominant, generalized connective tissue disease, affecting one in 2,500-5,000 persons [1-7]. It is caused by mutations in the gene encoding for neurofibromin, which has an essential role in vascular endothelial cells [1,2,4,7]. Vascular manifestations of NF1 have been recognized for over 50 years, but complications have often been underestimated [1,5,8]. Along with Ehlers-Danlos syndrome type IV and polycystic kidney disease, NF1 is often mentioned as a systemic heritable disorder associated with intracranial aneurysms [1]. With an incidence of 2-3.6%, this aspect of the disease is rarely encountered and often unrecognized in clinical practice [4,5]. The literature is not helpful in assessing the natural history of cerebrovascular lesions and widely accepted categorization does not exist, largely because of their small number [3]. We report a rare case of NF1 with a giant vertebral artery aneurysm and reversed cervical lordosis, which was treated successfully with embolization.

This case report was previously submitted as an abstract to the World Stroke Congress (WSC 2021), virtually held on October 28-29, 2021. This article was presented as a poster in the Sarawak Neurosurgery Update on June 27, 2021.

## Case presentation

**Figure 1:**
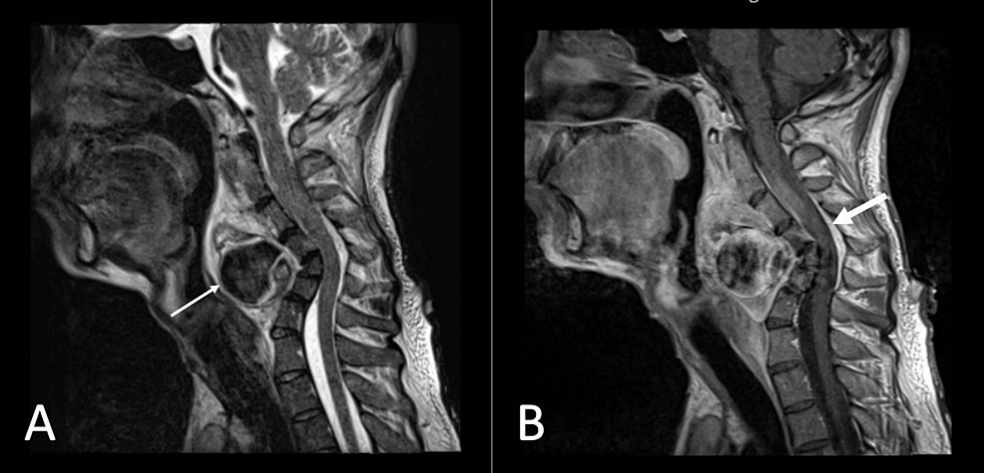
MRI neck. Neck MRI showed T2WI (A) heterogeneous extramedullary lesion (thin arrow) (retropharyngeal region), which was heterogeneously enhanced on post-contrast sagittal T1 (B) predominantly over C2 to C4 with moderate canal stenosis (thick arrow). MRI: magnetic resonance imaging; T2WI: T2 weighted image.

**Figure 2:**
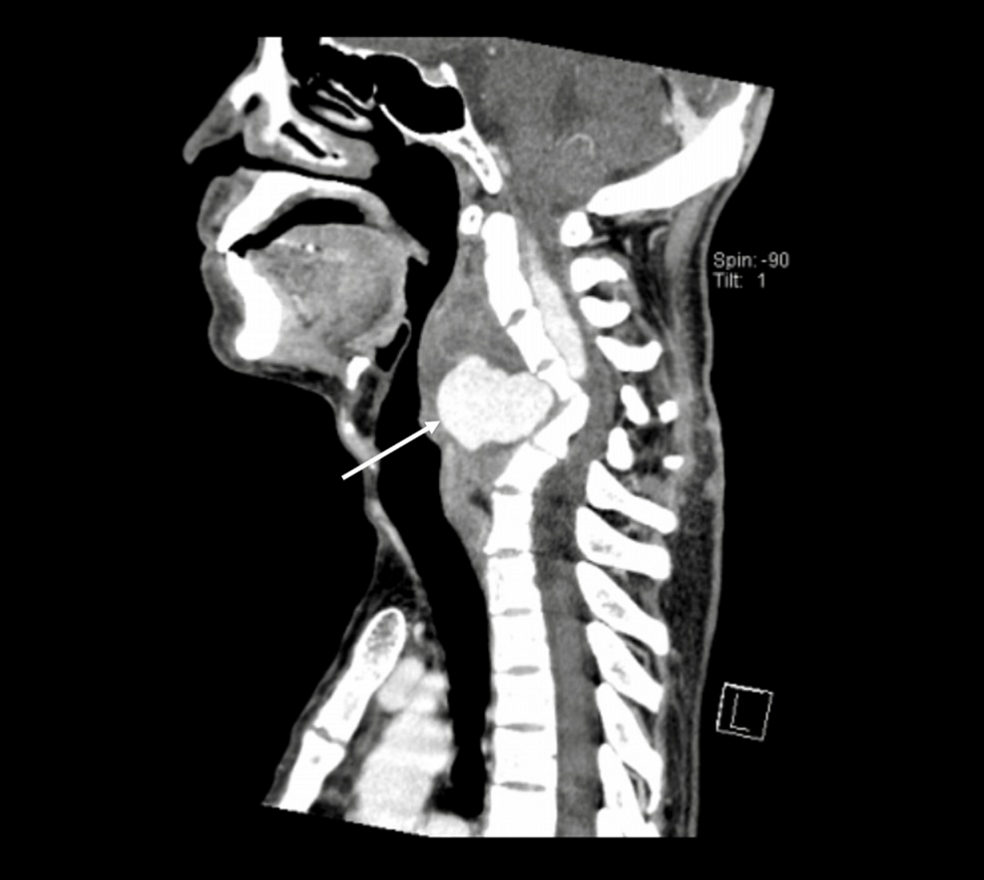
CTA vertebral artery. CTA vertebral artery in the sagittal plane showed the presence of a highly vascular lesion (thin arrow) suggestive of an aneurysm. CTA: computed tomography angiography.

**Figure 3:**
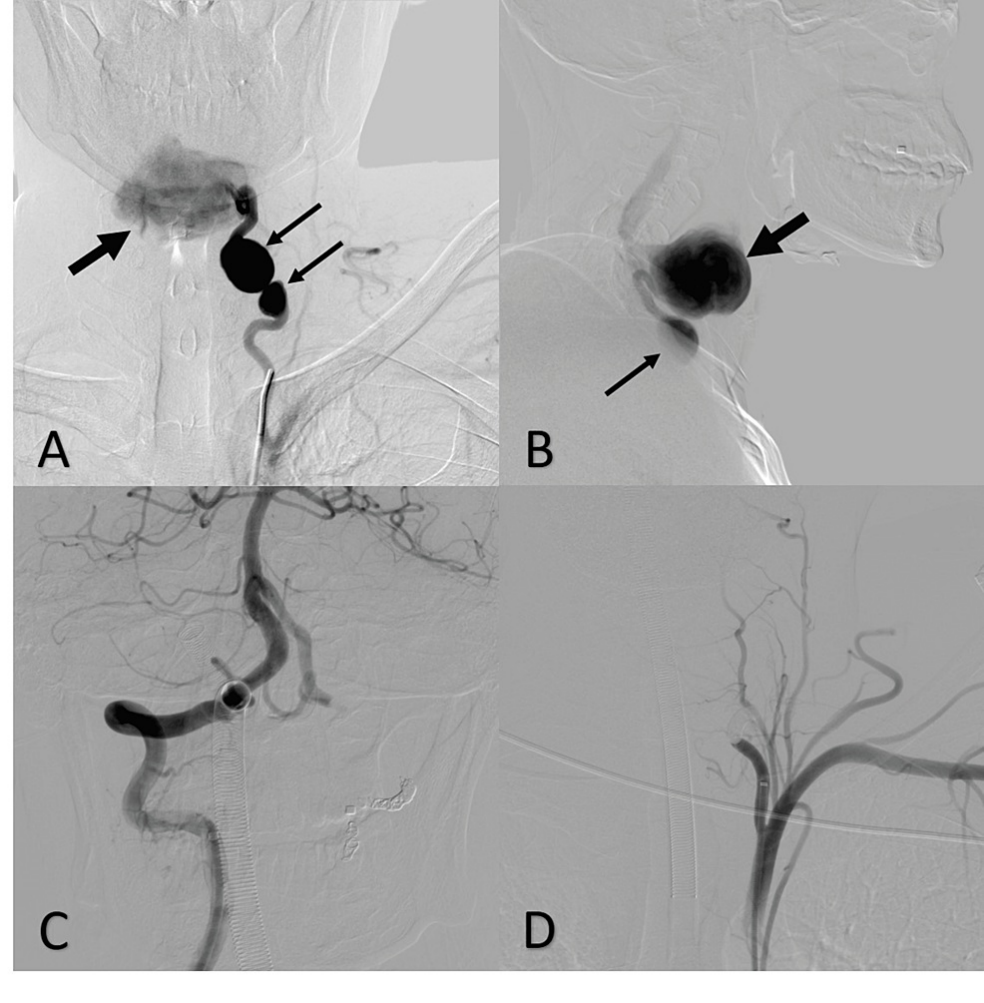
DSA vertebral artery. DSA of the left vertebral artery, pre-embolization anteroposterior (A) and lateral (B) images showed two fusiform aneurysms at the proximal left vertebral artery. A large pseudoaneurysm was distal to the fusiform aneurysms. Post-embolization DSA (C) and (D) showed no opacification of aneurysms (thin arrow: fusiform aneurysm, thick arrow: pseudoaneurysm). DSA: digital subtraction angiography.

A 31-year-old male presented with dysphagia, progressively worsening neck pain, and limited neck movement for a period of five months. He denied any preceding trauma. Examination revealed multiple cutaneous neurofibromas and a pulsating mass over the left side of the neck. He was otherwise neurologically intact. Magnetic resonance imaging (MRI) of the neck is suggestive of diffuse cutaneous and plexiform neurofibromas, associated with reversed cervical lordosis (Figure 1). MRI brain showed no brain parenchymal lesion and no evidence of acoustic neuroma. He was initially referred to us for excision of a neurofibroma. Computed tomography angiography (CTA) of the neck was ordered to ascertain the relationship of the mass to the surrounding vessels prior to surgery. However, CTA showed a highly vascular lesion (Figure 2) suggestive of the aneurysm at the retropharyngeal region causing compression to the adjacent oropharynx and hypopharynx and displacing bilateral carotid arteries and internal jugular veins laterally. Posteriorly, there is reverse cervical lordosis with remodeling and anterior scalloping of the C5 and C6 vertebrae. Digital subtraction angiography (DSA) confirmed the left vertebral artery fusiform aneurysm and pseudoaneurysm (Figure 3). Both aneurysms were successfully treated with embolization, and the patient fully recovered. Three months post-embolization, magnetic resonance angiography (MRA) revealed no flow within the left vertebral artery and no demonstrable arterial feeders to the mass. Follow-up physical examination and MRI showed no evidence of recurrent aneurysm, no arteriovenous malformation (AVM) or neurofibroma in the neck, and no progressive cervical kyphosis.

## Discussion

Arterial lesions in NF1 are characterized by stenosis, aneurysm, or fistula formation involving large and medium-sized arteries [1,3,8]. Children and young adults with NF1 may present vascular lesions predominantly in the abdominal aorta, renal arteries, internal carotid arteries, and vertebral arteries [2]. The majority of these lesions are clinically silent, hence frequently diagnosed in emergency situations [2]. Extracranial aneurysms may occur in the carotid, vertebral, coronary, mesenteric arteries, and parenchyma of the kidney [9,10]. Aneurysms of the extracranial vertebral artery are rare and almost all are traumatic; penetrating injuries, road traffic accidents, or sports injuries; seldom after chiropractic manipulations of the cervical spine [8,10]. Our patient never suffered prior cervical trauma in any form, hence vertebral artery aneurysm might be a manifestation of neurofibromatosis.

It has been suggested that patients with NF1 have some degree of vasculopathy and post-mortem examinations frequently show compression due to extrinsic tumors, arterial thickening, stenosis, and aneurysms [2,9]. Difficulty in obtaining specimens of affected vessels has limited the pathogenesis determination of aneurysm formation [1,9].

There are two main theories of pathomechanism leading to vasculopathy in neurofibromatosis. Some authors proposed that stenosis, post-stenotic dilatation, or aneurysm degeneration are consequences of vascular dysplasia [2-5,8,9]. Others claim that in larger arteries, the main pathology is due to direct vascular invasion from adjacent tumors such as schwannoma, neurofibroma, or neurofibrosarcoma leading to secondary fibrosis [2-5,8,9]. Previous evidence consisted of isolated case reports and small case series. In 1998, Saitoh et al. conducted immunohistological examinations of tissues involved in the ruptured brachial artery aneurysm of a NF patient and concluded that the origin of the proliferating tissue was not mesodermal dysplasia but neurofibroma occurring near or in the vessels [9].

As the vertebral artery aneurysm enlarges, it causes remodeling and scalloping of the adjacent vertebral body, widening of intervertebral foramina, and abnormality of facet joints, ligaments and back muscles, which promotes kyphosis [10]. Once kyphosis begins, the weight of the head can cause further curvature progress. Abnormality of ligaments and cervical spine may inadequately protect vertebral arteries from trauma. Hence, any minor trauma to the neck may precipitate a rapid increase in aneurysm size, or worst, a rupture.

Early presentation and a thorough physical examination are essential for diagnosis before rupture occurs. A pulsatile mass with bruits may be discovered during physical examination. The absence of both pulsation and bruit, however, does not rule out the possibility of an extracranial vertebral artery aneurysm. Aneurysms typically showed flow-void sign and high signal flow artifact on both non-contrasted T1 weighted image (T1WI) and T2WI, and enhancement on contrasted T1WI [11]. Onion skin appearance on non-contrasted T1WI with rim enhancement at the lumen margin may indicate a partially thrombosed aneurysm [11]. Historical and physical examination alone may render it difficult to differentiate between an aneurysm and an arteriovenous fistula, but in either situation, vertebral angiography is required for a definite diagnosis.

Although vascular integrity in neurofibromatosis is not severely affected compared with Ehlers-Danlos syndrome, surgery is not always an option and may be hazardous [1,2,8,9]. Surgical repair may be complicated by excessive bleeding, distortion of anatomic landmarks, and technical difficulties [1,2,8]. The risk of excessive bleeding is twofold; vessels are extremely brittle due to tumor invasion, and hypertrophied tissues are rich in small vessels [9].

Treatment of aneurysms associated with neurofibromatosis should not be resection and reconstruction of the affected vessels by autogenous vein grafting [9]. Surgical occlusion of aneurysms by direct ligation or clipping may be considered, though it is associated with a high rate of morbidity and mortality [8,9]. Poppen was believed to be the first to ligate the vertebrobasilar circulation for a reported vertebral artery lesion in 1945 [12]. With developments in endovascular techniques, permanent occlusion of the vertebral artery in the proximal posterior circulation is justified in selected patients with adequate collateral circulation [2,8,9,12].

The sacrifice of a parent artery is similar to surgical ligation and reduces complications associated with intra-aneurysmal obliteration [8]. However, endovascular occlusion might be a safer option that allows occlusion effects to be assessed prior to permanent obliteration [8]. This can avoid ischemic complications following inadequate collaterals to the posterior fossa [8].

## Conclusions

In conclusion, arterial manifestations in neurofibromatosis are unusual. As the majority of these lesions are clinically silent, vascular manifestations of NF1 have been underestimated in the past and often diagnosed in emergency situations. It is crucial for clinicians to maintain a high index of suspicion for the unusual presentation of the aneurysm in patients with neurofibromatosis as it may mimic clinical symptoms and radiological signs of neuromas. It is not recommended that aneurysmal pathology in a patient with neurofibromatosis be treated with vascular reconstruction. Recommended treatment is surgical or endovascular occlusion of the affected vessel.
